# Association between maternal fermented food consumption and child sleep duration at the age of 3 years: the Japan Environment and Children’s Study

**DOI:** 10.1186/s12889-022-13805-6

**Published:** 2022-08-06

**Authors:** Mariko Inoue, Narumi Sugimori, Kei Hamazaki, Kenta Matsumura, Akiko Tsuchida, Hidekuni Inadera, The Japan Environment and Children’s Study (JECS) Group, Michihiro Kamijima, Shin Yamazaki, Yukihiro Ohya, Reiko Kishi, Nobuo Yaegashi, Koichi Hashimoto, Chisato Mori, Shuichi Ito, Zentaro Yamagata, Takeo Nakayama, Hiroyasu Iso, Masayuki Shima, Hiroshige Nakamura, Narufumi Suganuma, Koichi Kusuhara, Takahiko Katoh

**Affiliations:** 1 Department of Public Health, Faculty of Medicine, University of Toyama, 2630 Sugitani, Toyama, 30-0194 Japan; 2 Toyama Regional Center for Japan Environment and Children’s Study, University of Toyama, 2630 Sugitani, Toyama, 930-0194 Japan; 3 Department of Public Health, Gunma University Graduate School of Medicine, Showa 3-39-22, Maebashi, Gunma 371-8511 Japan

**Keywords:** Probiotics, Child, Sleep, Circadian rhythm, Cheese, Health

## Abstract

**Background:**

Using cohort data from the Japan Environment and Children’s Study (JECS), we previously reported that the risk of sleep deprivation in 1-year-old children was reduced with a higher maternal intake of fermented foods, particularly miso. The present study, which evaluates children from the same cohort at 3 years of age, is a continuation of that work.

**Methods:**

After applying exclusion criteria to 104,062 records in the JECS dataset, we evaluated 64,200 mother-child pairs in which the child was 3 years old. We examined the association of the dietary intake of fermented foods during pregnancy with child sleep duration < 10 h at the age of 3 years.

**Results:**

Multivariable logistic regression analysis with the lowest quartile used as a reference revealed adjusted odds ratios (95% confidence intervals) for the second through fourth quartiles of 0.98 (0.90–1.06), 0.93 (0.85–1.01), and 0.85 (0.78–0.94) for cheese intake.

**Conclusions:**

The consumption of fermented foods during pregnancy is associated with reduced risk of sleep deprivation in 3-year-old children, albeit in a limited way.

**Supplementary Information:**

The online version contains supplementary material available at 10.1186/s12889-022-13805-6.

## Background

Children need a sufficient amount of good-quality sleep for healthy development. From the neonatal period to infancy and then early childhood, sleep patterns change with the child’s development. Short sleep duration has been reported to negatively affect physical and neurological development, including obesity in infancy and childhood [1, 2] and hyperactivity at 6 years of age [3]. Therefore, it is important to investigate the risk factors for sleep deprivation in children.

One of the factors that affect children is the diet of their mothers during pregnancy, which is recognized as a lifestyle factor. For example, probiotic-containing and fermented foods are thought to influence the gut microbiota [4] and have received considerable interest because they are associated with maternal health [5, 6] or, conversely, the development of diseases [7, 8], depending on the amount consumed. It has also been reported that children born by cesarean section are at higher risk of mental and developmental disorders, and one possible reason for this is that they are not exposed to their mother's gut bacteria at birth. With respect to the reported association between the microbiota at 1 year of age and neurocognitive development at 1 and 2 years of age [9, 10], maternal intake of fermented foods has been suggested to influence the normal development of children, especially sleep duration. In particular, the intestinal microbiota of children changes significantly from the neonatal period through infancy and weaning and stabilizes at around 3 years of year, reaching a composition similar to that of adults [11, 12]. In other words, vertical transmission of intestinal bacteria of maternal origin and maternal diet are predicted to affect the intestinal microbiota of children, but the association between maternal intake of fermented foods and children's sleep duration has not been examined on a large scale in epidemiological studies.

Against this background, we previously examined the association of maternal food intake preferences during pregnancy with infant sleep duration [13]. Specifically, using data from approximately 70,000 mother–infant pairs from a large cohort study, the Japan Environment and Children’s Study (JECS), we investigated the association between fermented food intake during pregnancy and infant sleep during the first postpartum year. We found that the higher the intake of fermented foods, especially miso soup, the more likely it is for the infant to sleep for at least 11 h. However, because the child’s brain grows exponentially until 2 years of age [14], it is important to clarify whether this association with fermented food persists beyond that point.

Therefore, to expand on our recent findings [13], we investigated whether maternal fermented food intake during pregnancy was associated with the sleep deprivation of children in the same cohort at 3 years of age.

## Methods

### Study population

The JECS protocol has been described elsewhere [15, 16]. In short, the JECS is a nationwide government-funded birth cohort study that aims to determine the associations of various environmental factors with child health and development. JECS participants are women residing in 15 regions of Japan who were enrolled during the first trimester of pregnancy between January 2011 and March 2014 [15, 16]. Follow-ups were conducted during the second or third trimester, at childbirth, and at 1 month postpartum during scheduled in-hospital checkups. Subsequent follow-ups were conducted at 12 and 36 months postpartum by mail.

**Fig. 1:**
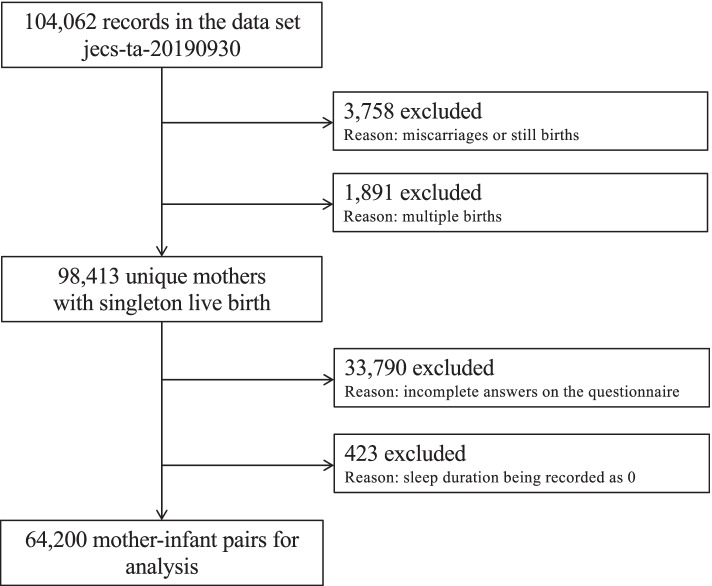
Flow diagram of the recruitment and exclusion process for participants

The present study analyzed the jecs-ta-20190930 dataset released in October 2019, which comprises 104,062 records obtained from a questionnaire-based survey of the participants. We excluded 3,758 cases that resulted in miscarriage or stillbirth and 1,891 cases of multiple births to focus on typical pregnancies (Fig. 1). Additionally, we also excluded 33,790 records because of incomplete responses to the questionnaire and 423 records for children whose sleep duration was recorded as 0, leaving 64,200 questionnaires with all data available for the final analysis.

### Ethics approval and consent to participate

The study protocol was approved by the Institutional Review Board on Epidemiological Studies of the Japanese Ministry of the Environment (authorization number: 100910001) and the ethics committees of all participating institutions. The JECS is conducted in accordance with the Declaration of Helsinki and all other national regulations, and written informed consent was obtained from parents/guardians of the participants whose age was below 16.

### Data assessment

#### Exposure

Dietary intake of fermented foods during pregnancy (from the discovery of pregnancy to the second or third trimester) was assessed using a food frequency questionnaire (FFQ) [17]. Fermented foods were foods such as cheese and yogurt, the preparation of which involves fermentation of food ingredients by microorganisms. This FFQ is a semi-quantitative instrument that assesses the average consumption of 171 food and beverage items. The FFQ includes four fermented foods: miso soup (made with miso, a Japanese traditional fermented seasoning), yogurt, cheese, and natto (Japanese fermented soybeans). The FFQ has not been validated specifically for pregnant women but has been validated in a large epidemiological study of adults in the general population and has already been used in a number of the JECS studies [18–20]. In this FFQ, participants were asked how often they consumed each food type and how much of it they consumed from learning of the pregnancy to the present. For miso soup, six frequency categories were used to record overall consumption frequency (from almost never to every day), nine frequency categories were used to record the daily consumption frequency (from < 1 time to ≥ 10 times), and five categories were used to report the taste of the miso soup (from very bland to very strong), which was taken to indicate the amount of miso in the soup. The daily intake (g/day) of miso was then calculated by multiplying the overall consumption frequency by the daily consumption frequency by a factor based on the reported taste. For the other three fermented foods—yogurt, cheese, and natto—the standard portion size for each food type was categorized as small (50% smaller than standard), medium (same as standard), or large (50% larger than standard). Nine frequency categories for each item were used to record consumption frequency (< 1 time/month to ≥ 7 times/day).

The daily intake of each of these three fermented foods was calculated by multiplying the consumption frequency by the standard portion size. Then, participants were categorized by quartile of intake amount (g/day) for each of the four fermented foods.

#### Outcome

To measure child sleep duration at 3 years after childbirth, parents were instructed to indicate when their child slept on the previous day. Parents marked the times when their child was asleep by drawing lines through boxes, indicating 30-min intervals, for the 24-h period beginning from 12:00 am at the start of the previous day.

Sleep duration of 10–13 h in a 24-h period is recommended for 3-year-old children by the United States National Sleep Foundation [21]. Therefore, we selected 10 h as the lower limit of the appropriate sleep duration and defined children sleeping less than this amount as having sleep deprivation.

#### Covariates

The covariates adjusted for were energy intake during pregnancy as assessed using the FFQ [17], maternal age during pregnancy, previous childbirth, body mass index (BMI) at 1 month after childbirth, maternal education level, annual household income during pregnancy, marital status at 6 months after childbirth, alcohol intake at 1 month after childbirth, smoking status at 1 month after childbirth, employment status at 1 year after childbirth, sex of the child, child attendance at nursery at 1 year after childbirth, the location where the child slept at night at 1 year after childbirth, birth weight, gestational age, consumption of dairy products at 3 years after childbirth, presence of any disease up to 3 years after childbirth, and date (month) of birth. These variables were categorized as in our previous study [13].

#### Statistical analyses

Unless otherwise stated, data are expressed as the mean ± standard deviation or median. Odds ratios (ORs) and 95% confidence intervals (95% CIs) for the risk of sleep deprivation according to each fermented food intake were calculated using logistic regression analysis, with each lowest quartile used as a reference. Adjusted ORs were calculated using all of the covariates described in the previous section, whereas crude ORs were calculated without adjustment for any covariates. In trend tests, categorical numbers were assigned to the quartile distributions for each fermented food intake and were treated as continuous variables. A two-sided *p*-value of < 0.05 was regarded as statistically significant. Analyses were performed using SAS version 9.4 (SAS Institute Inc., Cary, NC).

#### Additional analysis

To determine the association between overall fermented food intake during pregnancy and the sleep of their children at 3 years of age, we calculated the total score for quartiles of each of miso, yogurt, cheese, and natto during pregnancy, where the first quartile counted as 1 point, the second quartile as 2 points, and so forth. Thus, the score for the overall intake of fermented foods ranged from 4 to 16 points. The total score was also further categorized into quartiles. Analysis was likewise calculated using logistic regression analysis to obtain ORs and 95% CIs, setting the lowest quartile as the reference group.

## Results

Table 1 shows maternal characteristics according to the quartile of cheese intake during pregnancy. Participants with higher cheese intake were more likely to have high energy intake, to be older, to be multiparous, to have a normal weight (BMI: 18.5–<25), to have a higher education level, to have a higher household income, to be a nonsmoker, to be unemployed, and to send their child to a nursery. Table 2 shows maternal characteristics according to the quartile of miso intake during pregnancy. Participants with higher miso intake were more likely to be multiparous and nonsmokers, and less likely to send their child to a nursery. Tables S1 and S2 show maternal characteristics according to quartiles of yogurt and natto intake during pregnancy, which were similar to those for cheese intake.

**Table 1:** Characteristics according to quartile of maternal cheese intake during pregnancy (*n* = 64,200)

		Quartile of cheese intake									
		1 (low)		2		3		4 (high)		Total	
**Median intake of cheese, g/day**		0.0		1.3		4.3		10.0			
**Mean intake of energy, cal**		1,534		1,619		1,758		2,034		1,736	
**Age at childbirth, years**		30.9		31.4		31.9		32.3		31.6	
**Previous childbirth**	Nulliparous	7,090	(46.0)	7,187	(43.9)	6,748	(39.9)	5,993	(38.6)	27,018	(42.1)
	Multiparous	8,314	(54.0)	9,170	(56.1)	10,164	(60.1)	9,534	(61.4)	37182	(57.9)
**BMI (kg/m**^**2**^)	<18.5	783	(5.1)	779	(4.8)	859	(5.1)	797	(5.1)	3,218	(5.0)
	18.5–<25	11,979	(77.8)	13,068	(79.9)	13,752	(81.3)	12,635	(81.4)	51,434	(80.1)
	≥25	2,642	(17.2)	2,510	(15.4)	2,301	(13.6)	2,095	(13.5)	9,548	(14.9)
**Education level**	Junior high school or high school	6,175	(40.1)	5,591	(34.2)	4,852	(28.7)	3,972	(25.6)	20,590	(32.1)
	Technical junior college, technical/vocational college or associate degree	6,443	(41.8)	7,085	(43.3)	7,554	(44.7)	6,936	(44.7)	28,018	(43.6)
	Bachelor’s degree, postgraduate degree	2,786	(18.1)	3,681	(22.5)	4,506	(26.6)	4,619	(29.8)	15,592	(24.3)
**Annual household income, JPY**	<4 million	6,861	(44.5)	6,330	(38.7)	6,076	(35.9)	5,230	(33.7)	24,497	(38.2)
	4–<6 million	4,844	(31.5)	5,577	(34.1)	5,878	(34.8)	5,487	(35.3)	21,786	(33.9)
	≥6 million	3,699	(24.0)	4,450	(27.2)	4,958	(29.3)	4,810	(31.0)	17,917	(27.9)
**Marital status**	Married (including common law marriage)	15,107	(98.1)	16,142	(98.7)	16,728	(98.9)	15,375	(99.0)	63,352	(98.7)
	Divorced or widowed	130	(0.8)	112	(0.7)	90	(0.5)	73	(0.5)	405	(0.6)
	Others	167	(1.1)	103	(0.6)	94	(0.6)	79	(0.5)	443	(0.7)
**Alcohol intake**	Never	14,220	(92.3)	15,021	(91.8)	15,523	(91.8)	14,190	(91.4)	58,954	(91.8)
	Ex-drinker	618	(4.0)	692	(4.2)	788	(4.7)	703	(4.5)	2,801	(4.4)
	One to three times/month	382	(2.5)	438	(2.7)	426	(2.5)	442	(2.9)	1,688	(2.6)
	Once a week or more	184	(1.2)	206	(1.3)	175	(1.0)	192	(1.2)	757	(1.2)
**Smoking status**	Never	8,803	(57.2)	9,909	(60.6)	10,810	(63.9)	9,985	(64.3)	39,507	(61.5)
	Ex-drinker	3,472	(22.5)	3,786	(23.2)	3,871	(22.9)	3,728	(24.0)	14,857	(23.1)
	One to three times/month	2,413	(15.7)	2,130	(13.0)	1,827	(10.8)	1,464	(9.4)	7,834	(12.2)
	Once a week or more	716	(4.7)	532	(3.3)	404	(2.4)	350	(2.3)	2,002	(3.1)
**Employed**	No	7,752	(50.3)	8,395	(51.3)	8,953	(52.9)	8,471	(54.6)	33,571	(52.3)
	Yes	7,652	(49.7)	7,962	(48.7)	7,959	(47.1)	7,056	(45.4)	30,629	(47.7)
**Sex of the child**	Boy	7,504	(48.7)	8,124	(49.7)	8,171	(48.3)	7,667	(49.4)	31,466	(49.0)
	Girl	7,900	(51.3)	8,233	(50.3)	8,741	(51.7)	7,860	(50.6)	32,734	(51.0)
**Nursery**	No	5,354	(34.8)	5,887	(36.0)	6,248	(36.9)	5,927	(38.2)	23,416	(36.5)
	Yes	10,050	(65.2)	10,470	(64.0)	10,664	(63.1)	9,600	(61.8)	40,784	(63.5)
**Location where the baby sleeps at night**	In the parent's bed	12,933	(84.0)	13,719	(83.9)	14,294	(84.5)	13,019	(83.9)	53,965	(84.1)
	In a baby bed located in the parent's room	2,262	(14.7)	2,425	(14.8)	2,403	(14.2)	2,282	(14.7)	9,372	(14.6)
	In a baby bed located in a room other than the bedroom of his/her parents	152	(1.0)	145	(0.9)	161	(1.0)	173	(1.1)	631	(1.0)
	Other	57	(0.4)	68	(0.4)	54	(0.3)	53	(0.3)	232	(0.4)
**Birth weight, g**		3,018		3,028		3,034		3,035		3,029	
**Gestational weeks**		39.3		39.3		39.3		39.3		39.3	
**Eating dairy products**	Yes	14,971	(97.2)	15,953	(97.5)	16,502	(97.6)	15,164	(97.7)	62,590	(97.5)
	No	433	(2.8)	404	(2.5)	410	(2.4)	363	(2.3)	1,610	(2.5)
**Disease**	No	9,111	(59.2)	9,728	(59.5)	10,024	(59.3)	9,193	(59.2)	38,056	(59.3)
	Yes	6,293	(40.9)	6,629	(40.5)	6,888	(40.7)	6,334	(40.8)	26,144	(40.7)
**Birth month**	1	1,259	(8.2)	1,214	(7.4)	1,285	(7.6)	1,169	(7.5)	4,927	(7.7)
	2	1,063	(6.9)	1,126	(6.9)	1,187	(7.0)	1,056	(6.8)	4,432	(6.9)
	3	1,178	(7.7)	1,285	(7.9)	1,259	(7.4)	1,188	(7.7)	4,910	(7.6)
	4	1,223	(7.9)	1,254	(7.7)	1,297	(7.7)	1,179	(7.6)	4,953	(7.7)
	5	1,202	(7.8)	1,320	(8.1)	1,400	(8.3)	1,258	(8.1)	5,180	(8.1)
	6	1,202	(7.8)	1,258	(7.7)	1,368	(8.1)	1,243	(8.0)	5,071	(7.9)
	7	1,382	(9.0)	1,477	(9.0)	1,484	(8.8)	1,414	(9.1)	5,757	(9.0)
	8	1,555	(10.1)	1,660	(10.2)	1,669	(9.9)	1,618	(10.4)	6,502	(10.1)
	9	1,568	(10.2)	1,689	(10.3)	1,683	(10.0)	1,627	(10.5)	6,567	(10.2)
	10	1,433	(9.3)	1,567	(9.6)	1,631	(9.6)	1,416	(9.1)	6,047	(9.4)
	11	1,178	(7.7)	1,251	(7.7)	1,350	(8.0)	1,177	(7.6)	4,956	(7.7)
	12	1,161	(7.5)	1,256	(7.7)	1,299	(7.7)	1,182	(7.6)	4,898	(7.6)

**Table 2:** Characteristics according to quartile of maternal miso intake during pregnancy (*n* = 64,200)

		Quartile of miso intake									
		1 (low)		2		3		4 (high)		Total	
**Median intake of miso, g/day**		10.0		32.1		88.4		225.0			
**Mean intake of energy, cal**		1,620		1,697		1,752		1,868		1,736	
**Age at childbirth, years**		31.4		31.4		31.9		31.7		31.6	
**Previous childbirth**	Nulliparous	7,798	(47.6)	5,902	(43.0)	7,250	(40.3)	6,068	(37.7)	27,018	(42.1)
	Multiparous	8,603	(52.5)	7,818	(57.0)	10,738	(59.7)	10,023	(62.3)	37182	(57.9)
**BMI (kg/m**^**2**^)	<18.5	829	(5.1)	684	(5.0)	952	(5.3)	753	(4.7)	3,218	(5.0)
	18.5–<25	12,940	(78.9)	10,989	(80.1)	14,653	(81.5)	12,852	(79.9)	51,434	(80.1)
	≥25	2,632	(16.1)	2,047	(14.9)	2,383	(13.3)	2,486	(15.5)	9,548	(14.9)
**Education level**	Junior high school or high school	5,491	(33.5)	4,310	(31.4)	5,412	(30.1)	5,377	(33.4)	20,590	(32.1)
	Technical junior college, technical/vocational college or associate degree	7,062	(43.1)	6,041	(44.0)	7,921	(44.0)	6,994	(43.5)	28,018	(43.6)
	Bachelor’s degree, postgraduate degree	3,848	(23.5)	3,369	(24.6)	4,655	(25.9)	3,720	(23.1)	15,592	(24.3)
**Annual household income, JPY**	<4 million	6,579	(40.1)	5,360	(39.1)	6,511	(36.2)	6,047	(37.6)	24,497	(38.2)
	4–<6 million	5,374	(32.8)	4,624	(33.7)	6,233	(34.7)	5,555	(34.5)	21,786	(33.9)
	≥6 million	4,448	(27.1)	3,736	(27.2)	5,244	(29.2)	4,489	(27.9)	17,917	(27.9)
**Marital status**	Married (including common law marriage)	16,092	(98.1)	13,536	(98.7)	17,808	(99.0)	15,916	(98.9)	63,352	(98.7)
	Divorced or widowed	136	(0.8)	89	(0.7)	93	(0.5)	87	(0.5)	405	(0.6)
	Others	173	(1.1)	95	(0.7)	87	(0.5)	88	(0.6)	443	(0.7)
**Alcohol intake**	Never	14,963	(91.2)	12,574	(91.7)	16,586	(92.2)	14,831	(92.2)	58,954	(91.8)
	Ex-drinker	731	(4.5)	608	(4.4)	770	(4.3)	692	(4.3)	2,801	(4.4)
	One to three times/month	476	(2.9)	380	(2.8)	449	(2.5)	383	(2.4)	1,688	(2.6)
	Once a week or more	231	(1.4)	158	(1.2)	183	(1.0)	185	(1.2)	757	(1.2)
**Smoking status**	Never	9,851	(60.1)	8,525	(62.1)	11,221	(62.4)	9,910	(61.6)	39,507	(61.5)
	Ex-drinker	3,755	(22.9)	3,150	(23.0)	4,195	(23.3)	3,757	(23.4)	14,857	(23.1)
	One to three times/month	2,157	(13.2)	1,611	(11.7)	2,096	(11.7)	1,970	(12.2)	7,834	(12.2)
	Once a week or more	638	(3.9)	434	(3.2)	476	(2.7)	454	(2.8)	2,002	(3.1)
**Employed**	No	8,507	(51.9)	7,361	(53.7)	9,562	(53.2)	8,141	(50.6)	33,571	(52.3)
	Yes	7,894	(48.1)	6,359	(46.4)	8,426	(46.8)	7,950	(49.4)	30,629	(47.7)
**Sex of the child**	Boy	8,059	(49.1)	6,668	(48.6)	8,778	(48.8)	7,961	(49.5)	31,466	(49.0)
	Girl	8,342	(50.9)	7,052	(51.4)	9,210	(51.2)	8,130	(50.5)	32,734	(51.0)
**Nursery**	No	5,810	(35.4)	4,961	(36.2)	6,624	(36.8)	6,021	(37.4)	23,416	(36.5)
	Yes	10,591	(64.6)	8,759	(63.8)	11,364	(63.2)	10,070	(62.6)	40,784	(63.5)
**Location where the baby sleeps at night**	In the parent's bed	13,666	(83.3)	11,581	(84.4)	15,103	(84.0)	13,615	(84.6)	53,965	(84.1)
	In a baby bed located in the parent's room	2,489	(15.2)	1,960	(14.3)	2,662	(14.8)	2,261	(14.1)	9,372	(14.6)
	In a baby bed located in a room other than the bedroom of his/her parents	188	(1.2)	132	(1.0)	167	(0.9)	144	(0.9)	631	(1.0)
	Other	58	(0.4)	47	(0.3)	56	(0.3)	71	(0.4)	232	(0.4)
**Birth weight, g**		3,027		3,025		3,027		3,036		3,029	
**Gestational weeks**		39.3		39.3		39.3		39.2		39.3	
**Eating dairy products**	Yes	15,979	(97.4)	13,366	(97.4)	17,546	(97.5)	15,699	(97.6)	62,590	(97.5)
	No	422	(2.6)	354	(2.6)	442	(2.5)	392	(2.4)	1,610	(2.5)
**Disease**	No	9,676	(59.0)	8,074	(58.9)	10,740	(59.7)	9,566	(59.5)	38,056	(59.3)
	Yes	6,725	(41.0)	5,646	(41.2)	7,248	(40.3)	6,525	(40.6)	26,144	(40.7)
**Birth month**	1	1,140	(7.0)	1,030	(7.5)	1,418	(7.9)	1,339	(8.3)	4,927	(7.7)
	2	970	(5.9)	974	(7.1)	1,329	(7.4)	1,159	(7.2)	4,432	(6.9)
	3	1,059	(6.5)	993	(7.2)	1,491	(8.3)	1,367	(8.5)	4,910	(7.7)
	4	1,047	(6.4)	1,045	(7.6)	1,504	(8.4)	1,357	(8.4)	4,953	(7.7)
	5	1,138	(6.9)	1,094	(8.0)	1,551	(8.6)	1,397	(8.7)	5,180	(8.1)
	6	1,168	(7.1)	1,058	(7.7)	1,473	(8.2)	1,372	(8.5)	5,071	(7.9)
	7	1,450	(8.8)	1,207	(8.8)	1,595	(8.9)	1,505	(9.4)	5,757	(9.0)
	8	1,697	(10.4)	1,380	(10.1)	1,811	(10.1)	1,614	(10.0)	6,502	(10.1)
	9	1,877	(11.4)	1,433	(10.4)	1,694	(9.4)	1,563	(9.7)	6,567	(10.2)
	10	1,915	(11.7)	1,307	(9.5)	1,538	(8.6)	1,287	(8.0)	6,047	(9.4)
	11	1,510	(9.2)	1,142	(8.3)	1,264	(7.0)	1,040	(6.5)	4,956	(7.7)
	12	1,430	(8.7)	1,057	(7.7)	1,320	(7.3)	1,091	(6.8)	4,898	(7.6)

Compared with the excluded participants (*n* = 30,613), the included mothers (*n* = 64,200) were more likely to eat yogurt, cheese, and natto; to be older; to be married; to be a nonsmoker; to have a higher education level; to have a higher income; to be within the normal range of BMI; to be primiparas; and to have female infants with a heavier birth weight and longer gestational age.

ORs for children not meeting the 10-h sleep duration target were evaluated based on intake of miso, yogurt, cheese, and natto. In the cheese evaluation, ORs for inadequate sleep duration were significantly lower for children with mothers in the highest quartile of intake, and these associations were significant according to a trend test (Table 3).

**Table 3:** Odds ratios (95% confidence intervals) for risk of 3-year-old children sleeping less than 10 h per night according to quartile of maternal fermented food intake during pregnancy (*n* = 64,200)

	Quartile of fermented food intake				*p*-value
	1 (low)	2	3	4 (high)	for trend
Median intake of miso, g/day	10.0	32.1	88.4	225.0	
Total, n	16,401	13,720	17,988	16,091	
Cases, n	1,238	943	1,279	1,219	
Crude odds ratio	1.00 (Ref.)	0.90 (0.83–0.99)	0.94 (0.86–1.02)	1.00 (0.93–1.09)	0.809
Adjusted odds ratio	1.00 (Ref.)	0.91 (0.84–1.00)	0.95 (0.87–1.03)	1.00 (0.92–1.09)	0.863
Median intake of yogurt, g/day	8.0	25.7	60.0	120.0	
Total, n	16,933	14,471	14,210	18,586	
Cases, n	1,236	1,064	1,042	1,337	
Crude odds ratio	1.00 (Ref.)	1.01 (0.93–1.10)	1.01 (0.92–1.10)	0.98 (0.91–1.07)	0.689
Adjusted odds ratio	1.00 (Ref.)	1.04 (0.95–1.13)	1.04 (0.95–1.14)	1.02 (0.94–1.11)	0.674
Median intake of cheese, g/day	0.0	1.3	4.3	10.0	
Total, n	15,404	16,357	16,912	15,527	
Cases, n	1,190	1,231	1,212	1,046	
Crude odds ratio	1.00 (Ref.)	0.97 (0.90–1.06)	0.92 (0.85–1.00)	0.86 (0.79–0.94)	<0.001
Adjusted odds ratio	1.00 (Ref.)	0.98 (0.90–1.06)	0.93 (0.85–1.01)	0.85 (0.78–0.94)	<0.001
Median intake of natto, g/day	0.0	3.3	10.7	25.0	
Total, n	11,477	15,293	22,114	15,316	
Cases, n	869	1,151	1,557	1,102	
Crude odds ratio	1.00 (Ref.)	0.99 (0.91–1.09)	0.93 (0.85–1.01)	0.95 (0.86–1.04)	0.089
Adjusted odds ratio	1.00 (Ref.)	1.00 (0.91–1.10)	0.93 (0.86–1.02)	0.95 (0.86–1.05)	0.133

Adjusted for energy intake, maternal age during pregnancy, previous childbirth, body mass index (BMI) at 1 month after childbirth, maternal education level, annual household income, marital status at 6 months after childbirth, alcohol intake at 1 month after childbirth, smoking status at 1 month after childbirth, employment status at 1 year after childbirth, sex of the child, attendance at nursery (at 1 year of age), the location where the child slept at night (at 1 year of age), birth weight, gestational age, eating dairy products at 3 years of age, presence of any disease (up to 3 years of age), and date (month) of birth

In additional analyses, ORs were calculated for overall fermented food intake and children's sleep duration. The results showed that the OR for inadequate sleep duration was significantly lower for children whose mothers were in the highest quartile (adjusted OR 0.90, 95% CI 0.82–0.99), but not in the second (adjusted OR 0.99, 95% CI 0.91–1.08) or third (adjusted OR 0.94, 95% CI 0.87–1.03) quartile.

## Discussion

This study used data from 64,200 mother-child pairs from the JECS to determine the association of the dietary intake of fermented foods during pregnancy with less than 10 h of sleep among 3-year-old children. The results showed that cheese intake during pregnancy was associated with a significantly lower risk of sleep deprivation (< 10 h) among children of mothers in the fourth quartile compared with children of mothers in the first quartile. Miso intake was found to be associated with sleep duration in 1-year-old children [13] but not in 3-year-old children. These findings suggest that the effect of mothers’ consumption of fermented foods during pregnancy on their children’s sleep can continue to at least at 3 years of age.

The current results on the association between the maternal consumption of fermented foods during pregnancy and sleep duration in 3-year-old children are consistent with those from previous study [13]. It has already been reported that fermented foods positively affect the intestinal bacterial activity and growth [22].

In a randomized controlled trial with human participants, a group that consumed fermented foods such as yogurt and kimchi for 10 weeks had a greater variety of intestinal bacteria 4 weeks after the end of the study [23]. Animal experiments have shown that the gut microbiota, in addition to changing sleep-wake patterns and sleep quality, significantly alters gut metabolism, that the gut microbiota has a circadian rhythm, and that the intestinal bacteria exhibit circadian rhythms in composition and activity [24, 25]. It was also shown that mice without gut microbes have disrupted circadian rhythms compared with those with gut microbes [26]. In addition, maternal melatonin affects the fetus through the placenta [27, 28], and the gut microbiota is transferred to the infant at birth, causing changes in the infant’s gut microbiota [8]. The intestinal microbiota also reflects significant metabolic changes in the intestinal tract as well as changes in sleep-wake patterns and sleep quality [29]. Intestinal bacteria and hormones are thus expected to be closely related to sleep. Accordingly, fermented foods, intestinal flora, and hormones are closely related to sleep and the mother’s gut microbiota may have long-term effects on the child after birth.

The main strength of our study was the large sample size of over 60,000 mother–child pairs and the fact that the sample can be considered representative of mothers and toddlers in Japan, given that the JECS covers a wide geographic range across 15 regions. However, this study also has some limitations. Similar to the previous study [13], we did not directly investigate changes in intestinal microbiota. Another limitation is the reliance on maternal reports of child sleep duration. We observed that pregnant women who were well-educated and employed, and had higher income tended to have higher fermented food intake. To explain this, we speculated that these women likely recognized factors contributing to health and therefore tended to choose nutrient-rich options, such as fermented foods, more frequently than nutritionally unbalanced and/or nutrient-deficient options, such as junk food. The women’s health consciousness might affect the sleep duration of their children. In fact, the study found that cheese intake was associated with “health consciousness” factors such as BMI, education level, household income, and smoking status. Although we adjusted for these factors, “health consciousness” remained as a hidden factor independent of these other factors.

## Conclusions

In this study, 64,200 pairs of mothers and their children were surveyed to determine the association between the mothers' intake of fermented foods during pregnancy and their children's sleep duration at 3 years of age. The results showed that mothers who consumed more cheese during pregnancy had a reduced risk of their children sleeping less than 10 h per night.

## Supplementary Information

**Additional file 1.**

## Data Availability

This study’s data are unsuitable for public deposition due to ethical restrictions and the legal framework of Japan. It is prohibited by the Act on the Protection of Personal Information (Act No. 57 of 30 May 2003, amendment on 9 September 2015) to publicly deposit data containing personal information. Ethical Guidelines for Medical and Health Research Involving Human Subjects enforced by the Japan Ministry of Education, Culture, Sports, Science, and Technology and the Ministry of Health, Labour, and Welfare also restrict the open sharing of the epidemiological data. All inquiries about access to data should be sent to: jecs-en@nies.go.jp. The person responsible for handling inquiries sent to this e-mail address is Dr Shoji F. Nakayama, JECS Programme Office, National Institute for Environmental Studies.

## Abbreviations

BMI: body mass index
CI: confidence interval
FFQ: food frequency questionnaire
JECS: Japan Environment and Children’s Study
JPY: Japanese Yen
OR: odds ratio
