# Supplementary material for: Association between maternal fermented food consumption and child sleep duration at the age of 3 years: the Japan Environment and Children’s Study
Source: BMC Public Health. 2022 Aug 6;22:1504. doi: 10.1186/s12889-022-13805-6 (PMC9356427; doi:10.1186/s12889-022-13805-6)
Supplement: Supplementary file 1 — Additional file 1. [file 12889_2022_13805_MOESM1_ESM.docx]

**Table S1.** Characteristics according to quartile for yogurt intake during pregnancy in women (N = 64,200).

**Table S2.** Characteristics according to quartile for natto intake during pregnancy in women (N = 64,200)
